# Hexaaqua­zinc(II) 4,4′-(1,2-dihy­droxy­ethane-1,2-di­yl)dibenzoate monohydrate

**DOI:** 10.1107/S1600536810027972

**Published:** 2010-07-17

**Authors:** Da-Min Tian, Chong-Yu Shi, Cheng-Jun Hao

**Affiliations:** a Department of Chemistry and Chemical Engineering, Henan University of Urban Construction, Pingdingshan, Henan 467044, People’s Republic of China; b Zhongzhou University, Zhongzhou 450044, People’s Republic of China; c College of Chemistry and Chemical Engineering, Pingdingshan University, Pingdingshan 467000, People’s Republic of China

## Abstract

The title compound, [Zn(H_2_O)_6_](C_16_H_12_O_6_)·H_2_O, consists of one 4,4′-(1,2-dihy­droxy­ethane-1,2-di­yl)dibenzoate anion lying on an inversion centre, one [Zn(H_2_O)_6_]^2+^ dication lying on a mirror plane and one solvent water mol­ecule located on a mirror plane. The octahedral [Zn(H_2_O)_6_]^2+^ cations, solvent water mol­ecules and anions inter­act *via* O—H⋯O hydrogen bonds, forming a three-dimensional network.

## Related literature

For the architectures and potential application of polymeric coordination networks, see: Carlucci *et al.* (2003 ▶); Rosi *et al.* (2003 ▶). For the isostructural Mn complex, see: Hao & Cao (2010 ▶).
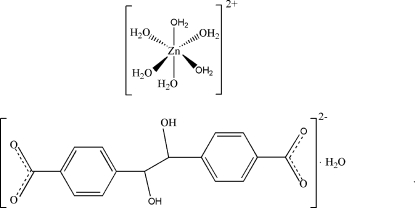


## Experimental

### 

#### Crystal data

[Zn(H_2_O)_6_](C_16_H_12_O_6_)·H_2_O
*M* *_r_* = 491.76
Monoclinic, 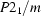

*a* = 6.0356 (9) Å
*b* = 20.508 (2) Å
*c* = 8.626 (1) Å
β = 104.141 (1)°
*V* = 1035.4 (2) Å^3^
*Z* = 2
Mo *K*α radiation
μ = 1.25 mm^−1^
*T* = 298 K
0.37 × 0.27 × 0.22 mm

#### Data collection

Bruker SMART 1000 CCD area-detector diffractometer
Absorption correction: multi-scan (*SADABS*; Bruker, 2007 ▶) *T*_min_ = 0.673, *T*_max_ = 0.759
5208 measured reflections
1877 independent reflections
1608 reflections with *I* > 2σ(*I*)
*R*_int_ = 0.035

#### Refinement

*R*[*F* ^2^ > 2σ(*F* ^2^)] = 0.068
*wR*(*F* ^2^) = 0.160
*S* = 1.26
1865 reflections
142 parameters
11 restraints
H-atom parameters constrained
Δρ_max_ = 0.65 e Å^−3^
Δρ_min_ = −0.45 e Å^−3^

### 

Data collection: *SMART* (Bruker, 2007 ▶); cell refinement: *SAINT* (Bruker, 2007 ▶); data reduction: *SAINT*; program(s) used to solve structure: *SHELXS97* (Sheldrick, 2008 ▶); program(s) used to refine structure: *SHELXL97* (Sheldrick, 2008 ▶); molecular graphics: *SHELXTL* (Sheldrick, 2008 ▶); software used to prepare material for publication: *SHELXTL*.

## Supplementary Material

Crystal structure: contains datablocks I, global. DOI: 10.1107/S1600536810027972/jh2181sup1.cif

Structure factors: contains datablocks I. DOI: 10.1107/S1600536810027972/jh2181Isup2.hkl

Additional supplementary materials:  crystallographic information; 3D view; checkCIF report

## Figures and Tables

**Table 1:** Hydrogen-bond geometry (Å, °)

*D*—H⋯*A*	*D*—H	H⋯*A*	*D*⋯*A*	*D*—H⋯*A*
O5*W*—H9*W*⋯O2^i^	0.84	1.94	2.774 (8)	177
O4*W*—H8*W*⋯O3^ii^	0.84	2.10	2.856 (7)	150
O4*W*—H7*W*⋯O5*W*	0.84	2.26	3.025 (9)	152
O3*W*—H5*W*⋯O2^iii^	0.84	2.65	3.306 (7)	136
O3—H3⋯O1^iv^	0.82	1.99	2.810 (7)	175
O1*W*—H2*W*⋯O3*W*^v^	0.84	1.94	2.770 (9)	172
O1*W*—H1*W*⋯O5*W*^v^	0.84	1.97	2.725 (10)	150
O2*W*—H3*W*⋯O1^vi^	0.84	2.02	2.810 (7)	155
O2*W*—H4*W*⋯O2^iii^	0.84	1.83	2.663 (7)	169
O3*W*—H5*W*⋯O1^iii^	0.84	1.87	2.699 (6)	169

Symmetry codes: (i) 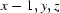; (ii) 
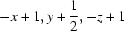
; (iii) 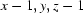; (iv) 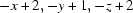; (v) 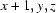; (vi) 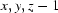.

**Fig. 1.**
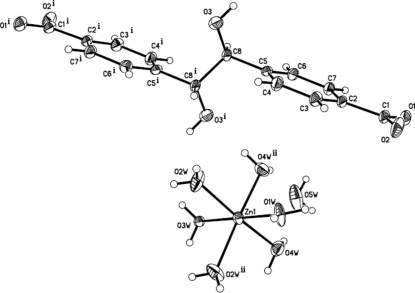
The structure of the title compound, showing the atomic numbering scheme. Non-H atoms are shown with 30% probability displacement ellipsoids (H atoms are omitted for clarity). [Symmetry codes: (i) 1-x, 1-y, 1-z; (ii) x, 1.5-y, z.]

**Fig. 2.**
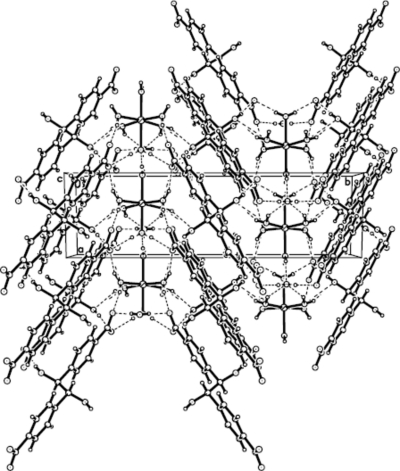
View of the three-dimensional network constructed by O—H···O hydrogen bonding interactions(the H atoms is not shown in the picture for clarity)

## supplementary crystallographic information

### Comment

Current interest in polymeric coordination networks is rapidly expanding for their intriguing architectures (Carlucci *et al.*, 2003) and potential applications (Rosi *et al.*, 2003). thus, we have reacted the ligand with Zn(NO3)_2_ under hydrothermal conditions to obtain new metal-organic complexes and the structure will be reported here.

As illustrated in figure 1, the title compound (C_16_H_12_O_6_)[Zn6H_2_O]. H_2_O is isostructural to a Mn complex based on the same ligand (Hao *et al.*, 2010), containing one 4,4'-(1,2-dihydroxyethane-1,2-diyl)dibenzoate anions ligand, one [Zn6H_2_O]^2+^ dicationic complex and a solvent water molecule, The carboxyl group lies in the plane of the benzene ring as indicated by the  O1—C1—C2—C3 and O2—C1—C2—C7 torsion angles of -0.2 (10) ° and 176.8 (6) °, and the two benzene rings are parallel to each other. In the crystal packing, a three-dimensional network was stabilized by a wide range of O—H···O hydrogen bonds involving the [Mn6H_2_O]^2+^ cations, 4,4'-(1,2-dihydroxyethane-1,2-diyl)dibenzoate anions and solvent water molecules.

### Experimental

A mixture of Zn(NO3)_2_ (0.1 mmol, 0.02 g) and 1,2-diol-1,2-bis(4-Carboxyphenyl) (0.1 mmol, 0.03 g) and 10 ml of H_2_O was loaded in a 20 ml Telflon-lined stainless steel vessel and heated at 303k for 2 days. Colorless crystals were obtained when the solution was cooled to room temperature slowly.

### Refinement

Carbon and nitrogen bound H atoms were placed at calculated positions and were treated as riding on the parent C or N atoms with C—H = 0.93 Å, N—H = 0.86 Å, and with *U*_iso_(H) = 1.2 *U*_eq_(C, N). H atoms of water molecule were located in a difference Fourier map and refined as riding, with Uiso(H) = 1.2Ueq(O)

### Crystal data

**Table:**

[Zn(H_2_O)_6_](C_16_H_12_O_6_)·H_2_O	*F*(000) = 512
*M**_r_* = 491.76	*D*_x_ = 1.577 Mg m^−^^3^
Monoclinic, *P*2_1_/*m*	Mo *K*α radiation, λ = 0.71073 Å
Hall symbol: -P 2yb	Cell parameters from 2215 reflections
*a* = 6.0356 (9) Å	θ = 2.5–24.0°
*b* = 20.508 (2) Å	µ = 1.25 mm^−^^1^
*c* = 8.626 (1) Å	*T* = 298 K
β = 104.141 (1)°	Block, colourless
*V* = 1035.4 (2) Å^3^	0.37 × 0.27 × 0.22 mm
*Z* = 2	

### Data collection

**Table:**

Bruker SMART 1000 CCD area-detector diffractometer	1877 independent reflections
Radiation source: fine-focus sealed tube	1608 reflections with *I* > 2σ(*I*)
graphite	*R*_int_ = 0.035
φ and ω scans	θ_max_ = 25.0°, θ_min_ = 2.0°
Absorption correction: multi-scan (*SADABS*; Bruker, 2007)	*h* = −7→7
*T*_min_ = 0.673, *T*_max_ = 0.759	*k* = −22→24
5208 measured reflections	*l* = −9→10

### Refinement

**Table:**

Refinement on *F*^2^	Primary atom site location: structure-invariant direct methods
Least-squares matrix: full	Secondary atom site location: difference Fourier map
*R*[*F*^2^ > 2σ(*F*^2^)] = 0.068	Hydrogen site location: inferred from neighbouring sites
*wR*(*F*^2^) = 0.160	H-atom parameters constrained
*S* = 1.25	*w* = 1/[σ^2^(*F*_o_^2^) + (0.*P*)^2^ + 7.6103*P*] where *P* = (*F*_o_^2^ + 2*F*_c_^2^)/3
1865 reflections	(Δ/σ)_max_ < 0.001
142 parameters	Δρ_max_ = 0.65 e Å^−^^3^
11 restraints	Δρ_min_ = −0.45 e Å^−^^3^

### Special details

**Table:**

Geometry. All esds (except the esd in the dihedral angle between two l.s. planes) are estimated using the full covariance matrix. The cell esds are taken into account individually in the estimation of esds in distances, angles and torsion angles; correlations between esds in cell parameters are only used when they are defined by crystal symmetry. An approximate (isotropic) treatment of cell esds is used for estimating esds involving l.s. planes.
Refinement. Refinement of F^2^ against ALL reflections. The weighted R-factor wR and goodness of fit S are based on F^2^, conventional R-factors R are based on F, with F set to zero for negative F^2^. The threshold expression of F^2^ > 2sigma(F^2^) is used only for calculating R-factors(gt) etc. and is not relevant to the choice of reflections for refinement. R-factors based on F^2^ are statistically about twice as large as those based on F, and R- factors based on ALL data will be even larger.

### Fractional atomic coordinates and isotropic or equivalent isotropic displacement parameters (Å^2^)

**Table:**

	*x*	*y*	*z*	*U*_iso_*/*U*_eq_	
Zn1	0.64994 (18)	0.7500	0.45837 (12)	0.0273 (3)	
O1	1.0813 (8)	0.6420 (2)	1.2091 (6)	0.0434 (13)	
O2	1.3273 (9)	0.6522 (3)	1.0577 (6)	0.0551 (15)	
O3	0.6711 (9)	0.4276 (2)	0.5239 (6)	0.0513 (15)	
H3	0.7356	0.4058	0.6016	0.077*	
C1	1.1427 (12)	0.6326 (3)	1.0801 (9)	0.0390 (18)	
C2	0.9861 (11)	0.5948 (3)	0.9467 (8)	0.0323 (16)	
C3	1.0476 (12)	0.5830 (3)	0.8047 (9)	0.0401 (18)	
H3A	1.1838	0.6000	0.7900	0.048*	
C4	0.9090 (12)	0.5462 (3)	0.6841 (9)	0.0424 (18)	
H4	0.9515	0.5390	0.5890	0.051*	
C5	0.7052 (11)	0.5202 (3)	0.7064 (8)	0.0355 (17)	
C6	0.6432 (12)	0.5318 (3)	0.8475 (8)	0.0384 (17)	
H6	0.5071	0.5147	0.8624	0.046*	
C7	0.7824 (12)	0.5687 (3)	0.9672 (8)	0.0379 (17)	
H7	0.7392	0.5761	1.0621	0.046*	
C8	0.5501 (12)	0.4796 (3)	0.5750 (8)	0.0382 (17)	
H8	0.4248	0.4616	0.6155	0.046*	
O1W	0.9972 (11)	0.7500	0.5576 (8)	0.051 (2)	
H2W	1.0963	0.7500	0.5043	0.076*	
H1W	1.0565	0.7500	0.6564	0.076*	
O2W	0.6821 (8)	0.6753 (3)	0.3037 (6)	0.0552 (16)	
H3W	0.7949	0.6545	0.2879	0.083*	
H4W	0.5702	0.6735	0.2233	0.083*	
O3W	0.2891 (10)	0.7500	0.3548 (7)	0.0258 (13)	
H5W	0.2368	0.7168	0.3015	0.039*	
O4W	0.5898 (9)	0.8184 (2)	0.6248 (6)	0.0453 (13)	
H7W	0.5008	0.8129	0.6846	0.068*	
H8W	0.5569	0.8518	0.5677	0.068*	
O5W	0.3294 (15)	0.7500	0.8358 (10)	0.090 (4)	
H9W	0.3342	0.7207	0.9046	0.135*	

### Atomic displacement parameters (Å^2^)

**Table:**

	*U*^11^	*U*^22^	*U*^33^	*U*^12^	*U*^13^	*U*^23^
Zn1	0.0228 (6)	0.0323 (6)	0.0253 (6)	0.000	0.0031 (4)	0.000
O1	0.033 (3)	0.043 (3)	0.046 (3)	−0.001 (2)	−0.007 (2)	−0.011 (2)
O2	0.030 (3)	0.066 (4)	0.061 (4)	−0.012 (3)	−0.004 (2)	−0.025 (3)
O3	0.053 (3)	0.032 (3)	0.056 (3)	0.003 (2)	−0.010 (3)	−0.006 (2)
C1	0.030 (4)	0.030 (4)	0.045 (4)	0.007 (3)	−0.013 (3)	−0.011 (3)
C2	0.026 (4)	0.026 (3)	0.036 (4)	0.005 (3)	−0.010 (3)	−0.007 (3)
C3	0.024 (4)	0.038 (4)	0.050 (5)	0.000 (3)	−0.006 (3)	−0.010 (3)
C4	0.039 (4)	0.039 (4)	0.042 (4)	0.005 (3)	−0.005 (3)	−0.011 (3)
C5	0.028 (4)	0.020 (3)	0.045 (4)	0.002 (3)	−0.016 (3)	−0.004 (3)
C6	0.035 (4)	0.029 (4)	0.042 (4)	−0.009 (3)	−0.007 (3)	−0.001 (3)
C7	0.035 (4)	0.033 (4)	0.038 (4)	0.000 (3)	−0.006 (3)	−0.007 (3)
C8	0.037 (4)	0.029 (4)	0.036 (4)	0.001 (3)	−0.016 (3)	−0.007 (3)
O1W	0.019 (4)	0.104 (7)	0.025 (4)	0.000	−0.003 (3)	0.000
O2W	0.023 (3)	0.075 (4)	0.059 (3)	0.011 (3)	−0.005 (2)	−0.036 (3)
O3W	0.022 (3)	0.025 (3)	0.029 (3)	0.000	0.002 (3)	0.000
O4W	0.051 (3)	0.043 (3)	0.040 (3)	−0.001 (2)	0.008 (2)	−0.012 (2)
O5W	0.054 (6)	0.180 (12)	0.036 (5)	0.000	0.011 (4)	0.000

### Geometric parameters (Å, °)

**Table:**

Zn1—O1W	2.061 (6)	C4—H4	0.9300
Zn1—O2W	2.071 (5)	C5—C6	1.379 (10)
Zn1—O2W^i^	2.071 (5)	C5—C8	1.528 (8)
Zn1—O4W^i^	2.101 (5)	C6—C7	1.386 (9)
Zn1—O4W	2.101 (5)	C6—H6	0.9300
Zn1—O3W	2.142 (6)	C7—H7	0.9300
O1—C1	1.270 (9)	C8—C8^ii^	1.535 (13)
O2—C1	1.243 (9)	C8—H8	0.9800
O3—C8	1.422 (8)	O1W—H2W	0.8387
O3—H3	0.8200	O1W—H1W	0.8393
C1—C2	1.512 (9)	O2W—H3W	0.8423
C2—C3	1.384 (10)	O2W—H4W	0.8423
C2—C7	1.392 (10)	O3W—H5W	0.8393
C3—C4	1.388 (9)	O4W—H7W	0.8385
C3—H3A	0.9300	O4W—H8W	0.8383
C4—C5	1.396 (10)	O5W—H9W	0.8396
			
O1W—Zn1—O2W	91.25 (19)	C5—C4—H4	120.2
O1W—Zn1—O2W^i^	91.25 (19)	C6—C5—C4	119.6 (6)
O2W—Zn1—O2W^i^	95.3 (3)	C6—C5—C8	120.0 (7)
O1W—Zn1—O4W^i^	92.5 (2)	C4—C5—C8	120.5 (7)
O2W—Zn1—O4W^i^	90.3 (2)	C5—C6—C7	120.4 (7)
O2W^i^—Zn1—O4W^i^	173.1 (2)	C5—C6—H6	119.8
O1W—Zn1—O4W	92.5 (2)	C7—C6—H6	119.8
O2W—Zn1—O4W	173.1 (2)	C6—C7—C2	120.6 (7)
O2W^i^—Zn1—O4W	90.3 (2)	C6—C7—H7	119.7
O4W^i^—Zn1—O4W	83.8 (3)	C2—C7—H7	119.7
O1W—Zn1—O3W	179.9 (3)	O3—C8—C5	111.7 (6)
O2W—Zn1—O3W	88.69 (17)	O3—C8—C8^ii^	105.9 (7)
O2W^i^—Zn1—O3W	88.69 (17)	C5—C8—C8^ii^	111.8 (7)
O4W^i^—Zn1—O3W	87.55 (18)	O3—C8—H8	109.1
O4W—Zn1—O3W	87.55 (18)	C5—C8—H8	109.1
C8—O3—H3	109.5	C8^ii^—C8—H8	109.1
O2—C1—O1	123.4 (6)	Zn1—O1W—H2W	124.1
O2—C1—C2	117.7 (7)	Zn1—O1W—H1W	124.0
O1—C1—C2	118.9 (7)	H2W—O1W—H1W	111.9
C3—C2—C7	118.8 (6)	Zn1—O2W—H3W	133.3
C3—C2—C1	120.7 (7)	Zn1—O2W—H4W	112.4
C7—C2—C1	120.5 (6)	H3W—O2W—H4W	111.2
C2—C3—C4	121.0 (7)	Zn1—O3W—H5W	115.9
C2—C3—H3A	119.5	Zn1—O4W—H7W	125.1
C4—C3—H3A	119.5	Zn1—O4W—H8W	101.5
C3—C4—C5	119.7 (7)	H7W—O4W—H8W	112.0
C3—C4—H4	120.2		
			
O2—C1—C2—C3	−0.2 (10)	C4—C5—C6—C7	−0.4 (10)
O1—C1—C2—C3	−179.5 (6)	C8—C5—C6—C7	−179.8 (6)
O2—C1—C2—C7	176.8 (6)	C5—C6—C7—C2	0.3 (10)
O1—C1—C2—C7	−2.5 (10)	C3—C2—C7—C6	−0.3 (10)
C7—C2—C3—C4	0.4 (10)	C1—C2—C7—C6	−177.3 (6)
C1—C2—C3—C4	177.4 (6)	C6—C5—C8—O3	−127.4 (7)
C2—C3—C4—C5	−0.5 (11)	C4—C5—C8—O3	53.2 (8)
C3—C4—C5—C6	0.6 (10)	C6—C5—C8—C8^ii^	114.1 (9)
C3—C4—C5—C8	179.9 (6)	C4—C5—C8—C8^ii^	−65.3 (10)

Symmetry codes: (i) *x*, −*y*+3/2, *z*; (ii) −*x*+1, −*y*+1, −*z*+1.

### Hydrogen-bond geometry (Å, °)

**Table:**

*D*—H···*A*	*D*—H	H···*A*	*D*···*A*	*D*—H···*A*
O5W—H9W···O2^iii^	0.84	1.94	2.774 (8)	177
O4W—H8W···O3^iv^	0.84	2.10	2.856 (7)	150
O4W—H7W···O5W	0.84	2.26	3.025 (9)	152
O3W—H5W···O2^v^	0.84	2.65	3.306 (7)	136
O3—H3···O1^vi^	0.82	1.99	2.810 (7)	175
O1W—H2W···O3W^vii^	0.84	1.94	2.770 (9)	172
O1W—H1W···O5W^vii^	0.84	1.97	2.725 (10)	150
O2W—H3W···O1^viii^	0.84	2.02	2.810 (7)	155
O2W—H4W···O2^v^	0.84	1.83	2.663 (7)	169
O3W—H5W···O1^v^	0.84	1.87	2.699 (6)	169

Symmetry codes: (iii) *x*−1, *y*, *z*; (iv) −*x*+1, *y*+1/2, −*z*+1; (v) *x*−1, *y*, *z*−1; (vi) −*x*+2, −*y*+1, −*z*+2; (vii) *x*+1, *y*, *z*; (viii) *x*, *y*, *z*−1.
